# Role of the Glymphatic System in Alzheimer’s Disease and Treatment Approaches: A Narrative Review

**DOI:** 10.7759/cureus.63448

**Published:** 2024-06-29

**Authors:** Mansi Thipani Madhu, Ojas Balaji, Venkataramana Kandi, Jayashankar CA, Ganaraja V Harikrishna, Nirosha Metta, Vamsi Krishna Mudamanchu, Bhangdiya G Sanjay, Praful Bhupathiraju

**Affiliations:** 1 Internal Medicine, Vydehi Institute of Medical Sciences and Research Centre, Bangalore, IND; 2 Medicine, Vydehi Institute of Medical Sciences and Research Centre, Bangalore, IND; 3 Clinical Microbiology, Prathima Institute of Medical Sciences, Karimnagar, IND; 4 Neurology, National Institute of Mental Health and Neurosciences, Bangalore, IND; 5 Neurology, Vydehi Institute of Medical Sciences and Research Centre, Bangalore, IND

**Keywords:** brain health, lymphatic system, aqp4, physical exercise, sleep, neurogenerative diseases, aquaporins, alzheimer’s disease, glymphatic system

## Abstract

Currently, there is unavailability of disease-modifying medication for Alzheimer's disease (AD), a debilitating neurological disorder. The pathogenesis of AD appears to be complex and could be influenced by the glymphatic system present in the central nervous system (CNS). Amyloid-beta (Aβ) and other metabolic wastes are eliminated from the brain interstitium by the glymphatic system, which encompasses perivascular channels and astroglial cells. Dysfunction of the glymphatic system, which could occur due to decreased aquaporin 4 (AQP4) expression, aging-related alterations in the human brain, and sleep disruptions, may contribute to the pathogenesis of AD and also accelerate the development of AD by causing a buildup of harmful proteins like Aβ. Promising approaches have been examined for reducing AD pathology, including non-pharmacological therapies that target glymphatic function, like exercise and sleep regulation. In addition, preclinical research has also demonstrated the therapeutic potential of pharmaceutical approaches targeted at augmenting AQP4-mediated glymphatic flow.

To identify the precise processes driving glymphatic dysfunction in AD and to find new treatment targets, more research is required. Innovative diagnostic and treatment approaches for AD could be made possible by techniques such as dynamic contrast-enhanced MRI, which promises to evaluate glymphatic function in neurodegenerative diseases. Treatment options for AD and other neurodegenerative diseases may be improved by comprehending and utilizing the glymphatic system's function in preserving brain homeostasis and targeting the mechanisms involved in glymphatic functioning. This review intends to enhance the understanding of the complex link between AD and the glymphatic system and focuses on the function of AQP4 channels in promoting waste clearance and fluid exchange.

## Introduction and background

One of the primary causes of dementia is Alzheimer's disease (AD), which has a lifetime prevalence of 5% to 7% and is currently one of the most deadly neurodegenerative diseases [1,2]. In this disease, harmful plaques are formed from abnormally expressed amyloid-beta (Aβ) and neurofibrillary tangles (NFTs) composed of phosphorylated tau protein. This may be due to the overactivation of microglia in the brain causing the secretion of neurotoxins and inflammatory factors [3,4]. Currently, disease-modifying treatments are unavailable to manage AD. Therefore, it is important to explore the disease mechanisms and develop therapeutic solutions against AD [5]. Although pharmacological interventions can postpone cognitive deterioration, non-pharmaceutical interventions need special emphasis [5].

The glymphatic system is the lymphatic system of the central nervous system (CNS). The glymphatic system comprises astroglial cells that consist of aquaporin (AQP) water channels through which cerebrospinal fluid (CSF) and interstitial fluid (ISF) exchange, forming a network of perivascular channels [6]. These channels allow waste to be removed and brain fluid to be cleared. When this clearance process is disrupted, harmful solutes like Aβ are not removed, which exacerbates the progression of diseases like AD [7-9].

The AQP4 water channels are mostly present in astrocytes and important for the exchange of fluids between CSF and ISF, which helps with the clearance of solutes [10]. The research found that AQP4 deficiency affects the brain parenchyma's ability to remove fluid, indicating its significance in preserving the fluid homeostasis of the brain [11]. While it is unknown what role AQP4 plays in neurodegeneration, reduced expression of AQP4 has been seen in animal models and AD patients [12,13]. The scope of AQP4 inhibitors as a treatment approach for neurological illnesses is being investigated by recent research [14,15].

This review focuses on the glymphatic system and its role in AD. It addresses the function of AQP4 in this system, the dysfunction of which leads to the pathogenesis of AD. The review discusses targeting the glymphatic system both pharmacologically and non-pharmacologically for therapeutic benefit in patients with AD.

## Review

The glymphatic system, a glia-dependent system of perivascular channels, has been identified in recent studies, dismissing previous views that there were no lymphatic vessels in the CNS. It is necessary to remove interstitial metabolic waste products, especially through the drainage of ISF and CSF. This is facilitated by the convective influx of CSF along the periarterial space [10]. Interstitial fluid is produced by metabolic substances and fluid released from capillaries and tissues, whereas CSF is thought to be secreted into the ventricles of the brain by the choroid plexus, circulated throughout the brain and spinal cord, and then is reabsorbed into blood vascular channels through structures known as arachnoid granulations [16]. The CSF is moved through perivascular gaps, which are found between the pia mater with astrocytic endfeet on the outside and the vessel wall on the inside. When CSF is produced, it enters the brain and circulates, causing the ISF in the tissue to convect toward the perivenous spaces, which are the spaces surrounding large, deep veins. The ISF is gathered in these perivenous spaces before it exits the brain and then travels to the cervical lymphatic system. From these perivenous gaps, CSF, ISF, and the byproducts of neural activity, including metabolic solutes and proteins, leave the brain parenchyma. It is noteworthy that ISF and CSF can exchange more easily with an arterial pulse wave and when there are AQP channels in the astrocytic endfeet [17].

**Figure 1:**
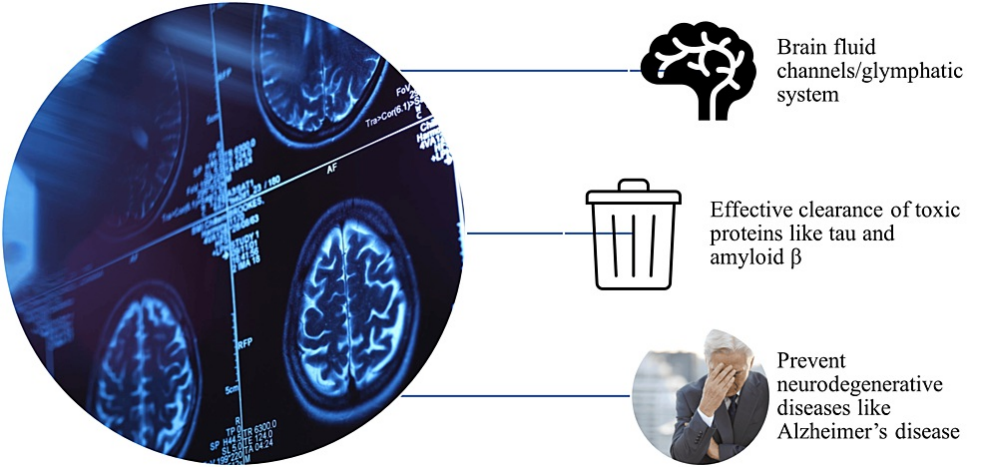
Role of the glymphatic system in preventing neurodegenerative diseases like Alzheimer's disease Image created by author Venkataramana Kandi

The proper functioning of the glymphatic system contributes to eliminating waste metabolites like lactate, proteins like tau and Aβ, and a range of contrast agents and tracers, as well as the pursuit of water and ionic balance [18,19]. It also serves other functions, including the transport of lipid signaling molecules and nutrients like glucose, lipids, amino acids, neurotransmitters, antigens, and immune cells, as well as the regulation of intracranial, ISF, and CSF pressure and the exchange of information through afferent and efferent immune pathways [10]. Therefore, efficient functioning of the glymphatic system is important to eliminate toxic protein accumulation in the brain and prevent neurodegenerative diseases like AD (Figure 1).

Aquaporins

Currently, there are 11 subtypes of AQP channels, which have been numbered from AQP0 to AQP10. While water is permeable through the channels numbered AQP0 to AQP6, water, urea, and glycerol are passable through AQP3, AQP7, and AQP8 channels. Further, purines, pyrimidines, and monocarboxylates are permeable through the AQP9 channel, also called the neutral solute channel [20]. 

In a study on rodents, it was noticed that AQP1 is mostly located in the epithelial cells of the choroid plexus, which is consistent with AQP1's suggested function in the production of CSF. The AQP4 subtype was initially identified as a CNS channel that primarily functions in brain fluid distribution [21]. The earliest evidence of AQP4 expression in the rat brain came from a hybridization study conducted in 1994 [22]. Some studies conducted in vitro on astrocyte cultures reveal that AQP4 regulates fluctuations in fluid volume, indicating that it plays an important part in maintaining extracellular homeostasis [23].

As a result of the development of glymphatic theory, AQP4's function in the clearance of fluid in the brain has received attention, and studies are being conducted in this direction. Previous research indicates the expression of AQP4 in ependymal and astrocyte cells and the lack of their expression in neurons [24]. There is sufficient evidence suggesting the crucial role of AQP4 in fluid movement throughout the brain [22].

The fluid and solutes must pass through AQP4 and the spaces between the astrocyte endfeet to reach the extracellular spaces. Besides that, convection-mediated fluid penetration is facilitated by the AQP4 channels, whose presence of high polarization permits ions and waste products released from the cells to cross the interstitium and reach the perivenous space [10]. In the white and gray matter, perivascular astrocytic endfeet surrounding the arterial structures show polarization of AQP4 [21,24].

**Figure 2:**
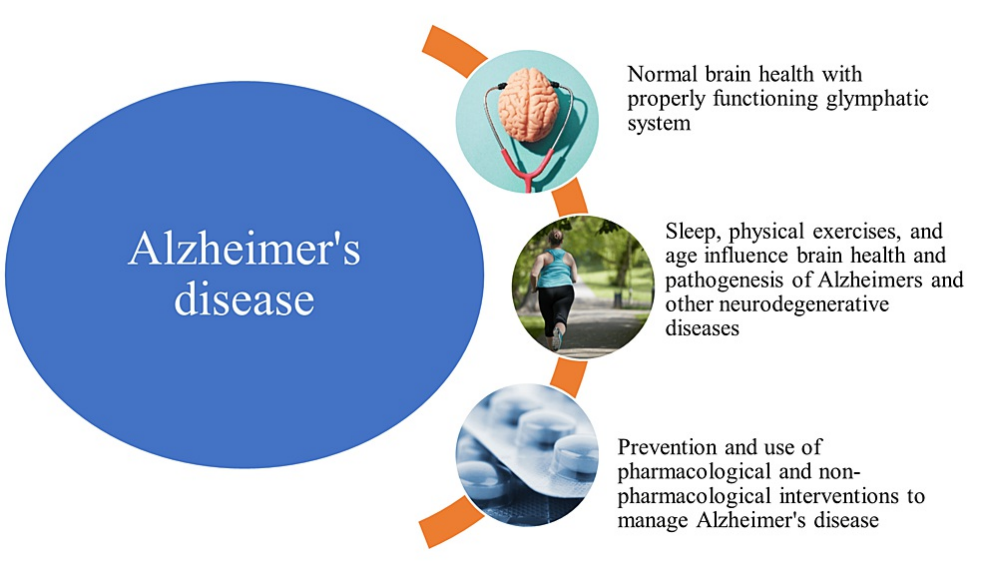
Pathogenesis, prevention, and management of Alzheimer's disease Image created by author Venkataramana Kandi

Furthermore, previous research performed on cortical slices has demonstrated that water flux through the astrocytic syncytium can be induced by brain vasopressin via activation of vasopressin 1a receptors, possibly through the mediation of water channels [23]. The results of previous studies comprehend regulatory processes to create therapeutic targets for neurodegenerative illnesses like AD. Additionally, there is increasing evidence of modifiable risk factors such as sleep and physical exercises influencing the pathogenesis of AD (Figure 2).

Glymphatic dysfunction in the pathogenesis of AD

Age, sleep deprivation, decreased activities of AQP4 channels, and vascular damage can all influence the glymphatic system, which can further predispose to AD.

Age

According to the available literature, the most prominent risk factor for dementia is aging [2]. Dynamic contrast-enhanced MRI (DCE-MRI) further suggests that human aging affects the glymphatic and meningeal lymphatic systems [25]. It is possible for the buildup of proteins in the extracellular space to occur before proteinopathy can develop as a result of compromise in regulatory clearance systems.

Reduced chemokine receptor type 7 (CCR7) expression and immunological activity in meningeal lymphatics have also been linked to aging. These factors can result in AQP4 expression being reduced and hence CSF glymphatic flow being compromised [26]. According to an investigation, reduced arterial pulsation and decreased expression of polarized AQP4 are present in middle-aged rodents, impairing CSF glymphatic influx and interstitial fluid tracer efflux [27].

Sleep Disturbances

Sleep disruption is another significant element that is also linked to aging and can impact individuals suffering from and having risk factors for AD. Elderly people frequently have sleep disorders such as reduced sleep length and quality, increased frequencies of waking up in the middle of sleep, an increase in the duration of falling asleep, and reduced rapid eye movement (REM) sleep [28]. These factors led to an increase in the activities of proteins tau and Aβ in the CSF of healthy individuals [29].

The findings of previous studies imply that aging and AD-associated sleep disorders may further contribute to toxic protein accumulation in the brain by affecting the glymphatic system. This observation is further strengthened by the fact that the glymphatic system is most active during sleep, and sleep deprivation significantly compromises glymphatic function [30, 31].

According to a DCE-MRI study, the aging human brain may have problems with the glymphatic pathway [32]. Other studies indicate that as people age, their cerebral arteries stiffen and their pulse amplitude diminishes, which results in a loss of glymphatic function [27,33,34]. Thus, more fluid and metabolites are retained, which block the perivascular pathways.

Decreased AQP4 Expression

There is sufficient evidence to suggest that depolarization of the AQP4 channels accelerates the course of AD. According to an investigation, perivascular AQP4 expression was correlated with lower levels of vascular Aβ deposition in patients with AD [35]. This indicates a potential link between toxic protein accumulation and the expression of AQP4. Additionally, patients with AD exhibit increased AQP4 immunoreactivity but significantly reduced expression of polarized AQP4 [36]. Moreover, there is a favorable correlation between cognitive function in AD patients and elevated perivascular diffusivity, a marker suggested for assessing lymphatic function by MRI [37].

It is noteworthy that CCR7 modulates the meningeal lymphatic immune function, as evident from the experiments on animal models of AD. It has been indicated that CCR7 knockout results in increased Aβ deposition and a decrease in cognitive functions [38]. These results further suggest a connection between hazardous protein buildup and decreased CNS waste clearance.

Glymphatic system as therapeutic target in AD

Decreased glymphatic flow has been demonstrated to encourage waste metabolite accumulation in the brain that results in neurodegenerative conditions such as AD. Thus, by altering variables that affect glymphatic function, such as consuming less alcohol and more omega-3 fatty acids, fasting occasionally, exercising, getting enough sleep, and controlling stress, neurodegeneration may be slowed or prevented [39-41].

Sleep Modulations

Sleep improves waste clearance in the CNS since the glymphatic system operates at its peak during sleep. Practicing sleep hygiene has been shown to improve sleep and its quality. Sleep hygiene guidelines recommend people engage in regular physical activity, minimize caffeine consumption in the late evenings, maintain consistent sleep and wake times, have sufficient light exposure upon waking up, practice meditation, stress management techniques like progressive muscle relaxation, follow a habitual routine before sleep time like reading a book, consuming food at least a couple of hours before sleep time, and cutting down on screen time (mobile phones, computer systems, and televisions) two hours before going to sleep [42].

Besides, enhancing non-REM (NREM) slow-wave sleep (SWS) is a potential AD therapeutic intervention. Research suggests that increasing slow-wave activity (SWA) in NREM sleep may help prevent AD by promoting the glymphatic system's ability to eliminate Aβ. Transcranial direct current and auditory closed-loop stimulation during NREM and SWS are techniques suggested to improve <1 hertz (Hz) NREM SWA. Transient direct current stimulation of <1Hz range helped memory consolidation in several groups. On the other hand, auditory closed-loop stimulation during NREM and SWS showed results in hippocampus-dependent memory consolidation [43].

Physical Exercise

Experimental animal studies have demonstrated the beneficial effects of physical activity on the functioning of the glymphatic system, leading to waste removal in the CNS. An investigation revealed that elderly mice using a running wheel considerably increased glymphatic influx and efflux of ISF drainage [44]. Based on research among young mice, exercise boosted glymphatic function and CSF influx in numerous brain areas [45]. Further research in this direction has been emerging, giving hope for the prevention and better management of neurodegenerative diseases like AD [46,47].

Pharmacological Intervention

Research is being carried out to develop pharmacologically active compounds to provide therapeutic benefits that can boost the function of the glymphatic system. Findings of previous studies have demonstrated that treatment with hypertonic saline or mannitol caused plasma hyperosmolarity in mice, which increased CSF influx into the brain and improved glymphatic function. Hyperosmolar therapy could correct glymphatic dysfunction and may enhance the delivery of antibodies against Aβ protein in the brain [48].

Researchers combined an antibody known as mAb158 and the vascular endothelial growth factor-C (mVEGF-C) using viral vectors to examine the therapeutic potential of meningeal lymphatics. The mice with AD pathology received this therapy in their CSF. The combined therapy synergistically affects the clearance of Aβ plaques and reduces inflammation. Additionally, older rats given mVEGF-C treatment demonstrated enhanced cognitive and lymphatic function. Subsequent studies on elderly transgenic mice with AD revealed that coadministration of antibodies and mVEGF-C reduces Aβ plaques. The gene expression study in the treated mice demonstrated effects, including treatment with aducanumab that enhanced synaptic structure pathways. Besides, treatment with mVEGF-C promoted dendritic growth, protein folding, and pathways related to learning and memory. The mVEGF-C therapy also activated interferon responses and antigen processing and presentation in the brain, pointing to possible mechanisms explaining the observed therapeutic effects [38].

A previous study investigated the role of n-3 polyunsaturated fatty acids (PUFAs) in clearing Aβ proteins. Experiments in mice utilizing oral fish oil delivery show that n-3 PUFAs increase Aβ clearance and protect against Aβ-induced damage. After Aβ injection, brain tissue imaging revealed that n-3 PUFAs prevent astrocyte activation and maintain AQP4 polarization in damaged areas. The AQP4 is a crucial part of the glymphatic system, as demonstrated by the reduced advantages in mice lacking AQP4 [41]. Experiments were conducted to examine the possibility of antiepileptic medications impeding the water transport characteristics of AQP4. These experiments support the theory that antiepileptic medications affect AQP4 [49].

Current research and future perspectives

The expression of AQP4 on astrocytic endfeet is necessary for the clearance of CSF, as multiple investigations have proven, and perivascular outflow of solutes like Aβ seems important for brain function and conditions such as AD. What is unknown is the exact method by which AQP4 channels mediate fluid transport between the interstitial and perivascular regions. Arterial pulsations may force fluid through AQP4, despite not transporting the solutes, including macromolecules [50].

A DCE-MRI evaluation, a non-invasive technique, of glymphatic drainage has demonstrated potential with an intrathecal infusion of gadolinium-based contrast agents [32,51,52]. Comprehending the role of waste clearance and toxic proteins in neurodegenerative diseases like AD, fully understanding the human glymphatic route, and defining the parameters affecting its operation can pave the way for developing therapeutic interventions. Many unanswered research questions and unexplored ideas for therapeutic approaches aimed at the glymphatic systems exist.

Experiments on mice looked at the function of spontaneous vasomotion in perivascular clearance. It was discovered that fluorescent dextran was removed from the ISF surrounding blood vessels in correlation with vasomotion. Higher clearance rates were exhibited when the amount of vasomotion was increased through visually triggered vascular responses. According to this research, solute drainage may be facilitated by low-frequency arteriolar oscillations, thereby preventing Aβ buildup in patients with AD. This may be achieved by focusing on natural vasomotion as a potential early therapeutic approach [53].

The MRI and the AQP4 facilitator N-[3-(benzyloxy)pyridin-2-yl] benzenesulfonamide (TGN-073) were used to confirm that improving AQP4 turnover pharmacologically boosted ISF circulation. According to this research, they also facilitated the glymphatic flow [54,55]. The in vitro inhibitory effects and in silico docking energies of 18 substances were examined for their influence on AQP4, and it has been shown that 2-(nicotinamide)-1,3,4-thiadiazole (TGN-020) inhibits AQP4 [56].

Most recently, AQP inhibitor AER-271 (N-(3,5-bis (trifluoromethyl)phenyl)-5-chloro-2-hydroxybenzamide) was identified as an efficient tool that can potentially manipulate the glymphatic system, as evidenced by the results on rodents [57]. Further modified TGN-020 with improved solubility was recently identified as an AQP4 inhibitor [58]. Despite the evidence, the development of drugs targeting AQPs is difficult due to several reasons [59]. The MRI was suggested as an alternative method to assess the functioning of the glymphatic system and evaluate the structure of perivascular spaces and parenchymal diffusibility [60].

The available research indicates that when AD worsens, the glymphatic system may become compromised, which could further exacerbate the disease. The meningeal and glymphatic systems have the potential to work together with anti-Aβ antibody therapy to improve brain waste clearance. More investigations are required to fully understand the human glymphatic route that defines the parameters affecting its operation. New prognostic and diagnostic tools and treatment targets may result from the glymphatic pathway's potential significance for maintaining human brain homeostasis.

## Conclusions

There is a potential interaction between the pathophysiology of AD and the glymphatic system mediated by AQP4 channels. The glymphatic system of the CNS is similar to the lymphatic system. It helps to keep the fluid homeostasis in the brain by removing waste products like Aβ. Deficits in the glymphatic system, like lowered expression of AQP4, aging-related disorders, and sleep disruptions, lead to the buildup of harmful proteins like Aβ and increase the risk of neurodegenerative diseases such as AD. Therapeutic approaches could be used to treat AD by focusing on the glymphatic system. Non-pharmacological therapies that improve glymphatic function, namely exercise and sleep regulation, present viable paths toward reducing AD pathology. Preclinical research and animal experiments indicate that pharmaceutical therapies that target AQP4 channels and glymphatic flow have promise for future therapeutic approaches. More investigations are necessary to discover new treatment targets and define the precise mechanisms driving glymphatic dysfunction in AD. It is possible to assess glymphatic function in neurodegenerative illnesses using techniques such as DCE-MRI, which may also lead to the creation of novel prognostic, diagnostic, and therapeutic strategies for AD and associated disorders. Ultimately, there is hope for treating neurodegenerative illnesses like AD by comprehending and utilizing the glymphatic system's function in preserving brain homeostasis.
